# Exploring the Roles of HERC2 and the NEDD4L HECT E3 Ubiquitin Ligase Subfamily in p53 Signaling and the DNA Damage Response

**DOI:** 10.3389/fonc.2021.659049

**Published:** 2021-03-31

**Authors:** Nicholas A. Mathieu, Rafael H. Levin, Donald E. Spratt

**Affiliations:** Gustaf H. Carlson School of Chemistry and Biochemistry, Clark University, Worcester, MA, United States

**Keywords:** HECT E3 ubiquitin ligases, WWP1, HERC2, HECW1, SMURF1, p53, DNA damage response

## Abstract

Cellular homeostasis is governed by the precise expression of genes that control the translation, localization, and termination of proteins. Oftentimes, environmental and biological factors can introduce mutations into the genetic framework of cells during their growth and division, and these genetic abnormalities can result in malignant transformations caused by protein malfunction. For example, p53 is a prominent tumor suppressor protein that is capable of undergoing more than 300 posttranslational modifications (PTMs) and is involved with controlling apoptotic signaling, transcription, and the DNA damage response (DDR). In this review, we focus on the molecular mechanisms and interactions that occur between p53, the HECT E3 ubiquitin ligases WWP1, SMURF1, HECW1 and HERC2, and other oncogenic proteins in the cell to explore how irregular HECT-p53 interactions can induce tumorigenesis.

## Introduction

Cell growth and division is controlled by the regulated synthesis and degradation of proteins that signal for and carry out the replication of DNA. This requires the timely expression and removal of proteins at specific checkpoints during the cell cycle to ensure proper cell division and homeostasis (1). When this delicate cellular equilibrium becomes imbalanced, unregulated cell division can occur and lead to the development of cancer. To protect against the formation of cancers, the cell has evolved an intricate network of proteins that work to recognize, target, and repair genetic abnormalities prior to its division. If significant cellular stress is recognized by these surveillance proteins, they will initiate a caspase cascade that activates lethal regulatory cell death (RCD) pathways, thereby preventing that cell from undergoing replication (2–9). Perhaps the most important protein involved in regulating these vital cellular activities is the tumor suppressor protein p53. Generally considered the “guardian of the genome”, p53 is a 43.7 kDa protein capable of undergoing more than 300 unique post translational modifications (i.e. phosphorylation (10), acetylation (11), methylation (12), SUMOylation (13), O-GlcNAcylation (14)) and interacts with a variety of proteins to dictate cellular fate following S-phase DNA duplication (15–20). One prominent PTM involved with regulating p53 activity under genotoxic and carcinogenic environments is ubiquitylation—a catalytic process that is carried out on p53 by select members of the homologous to E6AP *C-*terminus (HECT) E3 ubiquitin ligase family (21–24).

Over the past two decades, HECT-related cancer research has focused on the founding member of the HECT E3 ligase family, E6 associate protein (E6AP) (25–30). There are many studies that have cemented E6AP as a critical regulator of biochemical processes involved in the development of cervical and prostate cancer. For example, E6AP has been shown to interact with the human papilloma virus (HPV) protein E6 to target p53 for cellular degradation to produce unregulated cell division in the female cervical tissues (25). *In vivo* studies have also linked E6AP to metastatic forms of prostate cancer by acting to reduce tumor suppressor protein p27 expression levels in prostate gland cells (26, 30). Recent studies have also found that the members of the NEDD4L subfamily of the HECT E3 ubiquitin ligases—specifically WWP1, SMURF1, and HECW1—as well as the large HECT E3 ubiquitin ligase HERC2, are linked to the pathogenesis of prostate (31), lung (32–34), colon (35–38), breast (39, 40), thyroid (41), gastric (42), liver (43), oral (44), and ovarian cancers (45, 46).

This review aims to consolidate and examine the mounting literature on how additional members of the HECT E3 ubiquitin ligase family play integral roles in regulating DNA repair and p53 cellular activities. Here we explore and summarize the specific pathways, structures, and catalytic mechanisms used by WWP1, SMURF1, HECW1 and HERC2, and how their malfunction can result in oncogenesis. We also discuss developing a framework for future HECT-based cancer research that builds toward an improved overall understanding of oncogenic processes in the cell. Research on the interplay between these important protein networks will provide the necessary knowledge for developing novel treatment methods that can slow or even prevent the progression of HECT-dependent p53-related cancers.

## Ubiquitylation—A Brief Overview

Ubiquitylation involves the post-translational attachment of ubiquitin, a small 8.6 kDa protein, on to a substrate protein by an E1-E2-E3 enzymatic cascade (47–49). The human genome codes for two ubiquitin specific E1 enzymes (i.e. UBE1 and UBE1L2), 38 distinct E2 enzymes (ex. UBE2D3, UBE2L3, UBE2C, etc.) and over 1,000 unique E3 ligases (50). As ubiquitin is passed along the ubiquitylation signaling enzyme cascade (E1 to E2 to E3), the attachment of ubiquitin becomes more specific to ensure that the precise target protein is modified. This specificity from the ubiquitylation-signaling pathway can regulate various intracellular processes including protein turnover, cell cycle progression (51), apoptosis (52), cell differentiation and development (51), immune response and inflammation (53), intracellular trafficking (54), signal transduction (23), DNA transcription and repair (55), viral infection (53) and more. For the cell to carry out these processes, ubiquitin is first activated by an ubiquitin activating enzyme (E1; EC 6.2.1.45) through an ATP-dependent mechanism to form a thioester bond between the *C*-terminal carboxyl of ubiquitin and the catalytic cysteine of the E1. The ubiquitin is then transferred to an ubiquitin conjugating enzyme (E2; EC 2.3.2.23) *via* a trans-thiolation reaction to form a thioester bond between the *C*-terminus of ubiquitin and the conserved catalytic cysteine residue of the E2 (47, 56–58). The E2~ubiquitin complex next interacts with an ubiquitin ligase (E3) to properly coordinate the transfer of ubiquitin on to a specific lysine of the target substrate protein. Recent studies have also demonstrated that under specific cellular conditions the E3 ligases are able to catalyze the attachment of ubiquitin on to cysteine, threonine and *N*-terminal methionine residues of select target proteins (59–61). While the specific function of these alternative ubiquitin substrate attachments is not fully understood and requires further examination, they do add a further dimension to the permutations that can occur with the cellular ubiquitin machinery.

There are several different classes of E3 ubiquitin ligases found in humans that include the really interesting new gene finger domain-containing (RING; EC 2.3.2.27) (62), U-box (63), RING-between-RING (RBR; also known as RING-BRcat-Rcat; EC 2.3.2.31) (64) and HECT (E.C. 2.3.2.26) E3 ubiquitin ligases. The RING E3 ubiquitin ligases are the largest and most widely studied family of E3 ligases with over 600 members identified in the human genome (65). During ubiquitylation, these enzymes act as protein scaffolds that orient the E2~ubiquitin thiolester complex and target substrate to allow for efficient ubiquitin transfer (62, 66). In contrast, the RBRs catalyze substrate ubiquitylation by using a RING-like mechanism to coordinate an ubiquitin charged E2 cognate enzyme, followed by the formation of a HECT-like thiolester intermediate between ubiquitin and the enzyme’s Rcat domain to complete the ubiquitin cargo transfer onto the substrate (64, 67–69). Lastly, the HECT E3 ubiquitin ligases play a catalytic role in the final attachment of ubiquitin by forming a thiolester intermediate with its conserved catalytic cysteine prior to transferring the ubiquitin to a substrate protein (70–74).

In the context of p53 ubiquitylation, several HECT E3 ubiquitin ligases have been shown to play a role in the final attachment of ubiquitin to p53. The specific HECT E3 ubiquitin ligase that attaches ubiquitin onto p53 decides the isopeptide linkages formed in a mono-, multi mono- or polyubiquitin chain (i.e., linear *via N-*terminal M1, or K6, K11, K27, K29, K33, K48, and/or K63) to modulate p53 activity and dictate its cellular function (47–49). For example, a K48-linked polyubiquitin chain attached to p53 signals for p53 turnover by the 26S proteasome (47–49), while K63-linked polyubiquitin chains control p53 intracellular trafficking (75) and transcriptional regulation of the complex between p53 and the RING E3 ubiquitin ligase mouse double minute 2 (MDM2) (76). Recent studies have also demonstrated that the HECT E3 ubiquitin ligases SMURF1 and HERC2 can regulate the activity of p53 independent of their ubiquitylation activities (i.e., no ubiquitin transfer or chain formation) (77, 78).

## HECT E3 Ubiquitin Ligases—Important Enzymes in Oncogenesis

The HECT E3 ubiquitin ligase family is comprised of 28 enzymes that contain a characteristic ~350 residue catalytic HECT domain found near their *C*-termini (70, 79, 80). The HECT domain is bi-lobal, where the *N*-lobe (~250 residues) is responsible for recruiting and binding the E2~ubiquitin complex, while the *C*-lobe contains the absolutely conserved catalytic cysteine responsible for the ubiquitin transfer onto a target substrate (81, 82). Structures of the isolated HECT domains from different HECT family members have revealed unique conformational orientations for the *C*-lobe, with some showing the *C*-lobe in close proximity to the *N*-lobe while others showing a large distance of separation. These findings suggest that a flexible linker exists between the *N*-lobe and *C*-lobe of the HECT domain that allows for the free rotation of the *C*-lobe for accepting ubiquitin from the E2 and subsequently transferring it to a target substrate.

**Figure 1:**
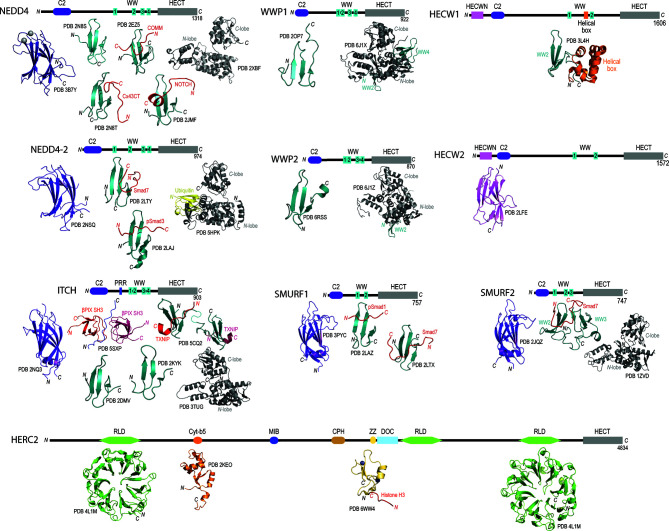
Domain architecture and catalogue of three-dimensional structures of domains for members for the NEDD4L subfamily and HERC2. Domain architecture schematics are based upon annotated boundaries on Uniprot. All of the published or publicly available 3D structures were visualized using PyMol. NEDD4 – C2 domain bound to calcium (purple and silver spheres; PDB 3B7Y), WW domain 1 (PDB 2N8S) (86), WW domain 2 (cyan) in complex with phosphorylated Cx43CT (red; PBD 2N8T) (86), WW domain 3 (cyan) in complex with COMM (red; PDB 2EZ5) (87), WW domain 4 (cyan) in complex with NOTCH (red; PDB 2JMF) (88), and HECT domain (grey; PDB 2XBF) (89). NEDD4-2 – C2 domain (purple; PDB 2NSQ), WW domain 2 (cyan) in complex with Smad7 (red; PDB 2LTY) (90), WW domain 3 in complex with phosphorylated Smad3 (PDB 2LAJ) (91) HECT domain in complex with ubiquitin (PDB 5HPK) (92). ITCH – C2 domain (purple; PDB 2NQ3), proline-rich region (green) with βPix SH3 domains (reds; PDB 5SXP) (93), WW domain 1 (cyan; PDB 2DMV), WW domain 2 (cyan; PDB 2KYK), WW domains 3 and 4 (cyan) in complex with TXNIP peptide (red; PDB 5CQ2) (94), and HECT domain (grey; PDB 3TUG). WWP1 – WW domain 4 (cyan; PDB 2OP7), and WW domains 2, 3 and 4 (cyan) with HECT domain (grey; PDB 6J1X) (95). WWP2 – WW domain 4 (cyan; PDB 6RSS) (96), and WW domains 2, 3 and 4 (cyan) with HECT domain (grey; PDB 6J1Z) (95). SMURF1 – C2 domain (purple; PDB 3PYC), WW domain 1 (cyan) in complex with phosphorylated Smad1 (red; PDB 2LAZ) (91), and WW domain 2 (cyan) in complex with Smad7 (red; PDB 2LTX) (90). SMURF2 – C2 domain (purple; PDB 2JQZ) (97), WW domains 2 and 3 (cyan) in complex with Smad7 (red; PDB 2KXQ) (98), and HECT domain (grey; PDB 1ZVD) (99). HECW1 – Helical box (orange) with WW domain 2 (cyan; PDB 3L4H). HECW2 – HECWN domain (pink; PDB 2LFE). HERC2 – RLD domain 1 (green; PDB 4L1M), Cyt-b5 domain (orange; PDB 2KEO), ZZ domain bound to zinc ions (purple and grey spheres) in complex with Histone H3 tail (red; PDB 6WW4) (100), RLD domain 3 (green; PDB 3KCI).

Apart from the highly conserved HECT domain, there is remarkable diversity in the protein-protein interaction domains found at the *N*-termini of members in the HECT family. Through the biochemical and structural distinction of these *N*-terminal domains, the family of 28 enzymes has been classified into three different HECT subfamilies i) neuronal precursor cell-expressed developmentally downregulated 4 (NEDD4), ii) HECT and RLD containing (HERC), and iii) “Other” (70, 83). Focusing specifically on the NEDD4 subfamily, each of the nine members have been shown to contain an *N*-terminal C2 calcium binding domain involved with binding phospholipids through a calcium dependent mechanism (84), and two, three or four tryptophan-tryptophan (WW) domains involved in recognition of substrates with proline-rich motifs (i.e., PPxY or PY) (85) (**Figure 1**). Additionally, NEDD4 family members HECT, C2 and WW-domain containing protein 1 (HECW1) and HECW2 contain a unique HECW1/2 *N*-terminal domain thought to be involved in substrate recognition but has yet to be documented in the literature. Another HECT E3 ubiquitin ligase member belonging to the HERC subfamily, HECT and RLD containing protein 2 (HERC2), illustrates the vast diversity in *N*-terminal interaction domains (**Figure 1**). The HERC2 *N*-terminal protein-protein interaction domains include three regulator of chromosome condensation 1-like domains (RLDs) that are suggested to be involved in chromatin binding, centrosome assembly and guanine nucleotide exchange (101, 102), a zinc finger (ZF) domain that is required for protein/DNA binding (103), a unique Cullin-7-PARC-HERC2 (CPH) domain predicted to be involved in binding to tetramerized p53 (104, 105), and a cytochrome-b5 like domain (cyt-b5) that binds to heme and acts as a redox potential interaction domain with electron transport like properties (106). HERC2 also contains a mind bomb (MIB) domain that is thought to be involved in regulating the Notch signaling pathway to ensure proper intercellular communication during embryonic stem cell differentiation (107) and a downregulated in ovarian cancer (DOC) domain, which is similar to the anaphase promoting complex (APC) and may have a role in in the ubiquitylation activity of HERC2 (108).

The variability at the *N*-terminal protein-protein interaction domains in members of the NEDD4 subfamily and HERC2 suggest that these enzymes bind and recognize a broad range of substrate proteins in the context of oncogenesis (70) (**Table 1**). For example, it has been shown that HECW1 (aka NEDL1), SMAD ubiquitylation regulatory factor 1 (SMURF1), WW-domain containing protein 1 (WWP1) and HERC2 each carryout unique interactions with p53 that involve either the direct K63 ubiquitylation of p53, as in the case of WWP1 (75) (89), and/or the formation of multiprotein enzymatic complexes that act to modulate p53 activity independent from HECT-dependent ubiquitylation (149). Collectively, these HECT-dependent interactions have been identified as critical regulators of p53 activity that impact apoptotic signaling (149), the transcription of p53 related genes (75), equilibrium of the MDM2-p53 feedback loop (105, 150), ataxia telangiectasia mutated (ATM) and ataxia telangiectasia and Rad3 related (ATR) dependent DNA double strand break responses (151), and other oncological-related cellular responses.

**Table 1:** Examples of experimentally observed protein-protein interaction of oncogenic proteins with HECT E3 ubiquitin ligases.

Oncogenic Protein	HECT E3 ubiquitin ligase	Experimental detection method	Region of interaction	References
Cellular tumor antigen p53 (p53, TP53)	E6AP	2H, 3H, CE, CL, IF, IP, ITC, MS, PD, SPR, UbA, X-ray	280-781 aa	(71, 79, 109–130)
	HECW1	IP		(77)
	WWP1	IP, PD, UbA		(75)
	HERC2	IP, PD	CPH (2547-2640 aa)	(105)
Cellular tumor antigen p63 (p63, TP63)	WWP1	IP, UbA		(131)
	ITCH	IF, IP, NMR	WW domains 1&2	(132–136)
	NEDD4	2H, IP, UbA		(137)
Cellular tumor antigen p73 (p73, TP73)	HECW2	IP, PD, UbA	WW domains 1&2	(138)
	ITCH	IP, TAP, UbA		(139)
Apoptosis-stimulating of p53 protein 2 (TP53BP2)	E6AP	2H, 3H, IP		(115)
	ITCH	IF, IP, MS, PD, TAP	WW domains 1-4	(140, 141)
Melanoma-associated antigen 12 (MAGE12)	E6AP	2H, 3H		(115)
Promyelocytic leukemia protein (PML, MYL TRIM19)	E6AP	IF, IP, UbA		(142)
Mouse double minute 2 homolog (MDM2)	NEDD4	MS, PD, UbA		(143)
Breast cancer type 1 susceptibility protein (BRCA1)	HERC2	IP, MS, UbA	HECT domain (4252-4834 aa)	(144)
BCL-2-antagonist/killer (BAK)	HERC1	IF, PLISA	BH3 domain	(145)
Large tumor suppressor 1 (LATS1)	ITCH	IP, MS, PD, UbA	WW domains 1-4	(146, 147)
Protein Kinase B (AKT)	ITCH	MS	Phosphorylation @ S257	(148)

**Detection methods**: 2H, yeast or mammalian-two hybrid; 3H, yeast or mammalian-three hybrid; CE, co-elution during chromatography purification; CL, chemical crosslinking; IF, immunofluorescence; IP, immunoprecipitation; ITC, isothermal titration calorimetry; MS, liquid chromatography or MALDI MS/MS; NMR, nuclear magnetic resonance; PD, pulldown using GST, His or MBP tag; PLISA, proximity ligation in situ assay; SPR, surface plasmon resonance; TAP, tandem affinity purification; UbA, ubiquitylation assay; X-ray, X-ray crystallography.

Given the HECT family’s diverse regulation of p53 coupled with the well-established role of p53 in maintaining proper cell division and DNA integrity, members of the HECT E3 ubiquitin ligase family are promising oncological drug targets where their structural and mechanistic interactions with p53 can potentially be directed to modulate p53 activity and elicit precise HECT-p53 dependent anti-cancer cellular responses.

## The NEDD4 HECT E3 Ubiquitin Ligases Play Diverse Roles in p53 Modulation

The NEDD4 subfamily has become increasingly important in the field of oncology, as various members have been found to upregulate and interact with important tumor suppressor network proteins such as p53. Here we describe how WWP1, HECW1 and SMURF1 regulate p53-dependent cellular functions.

### WWP1 Facilitates p53 Aggregation in the Cytoplasm in Response to p53 Overexpression

**Figure 2:**
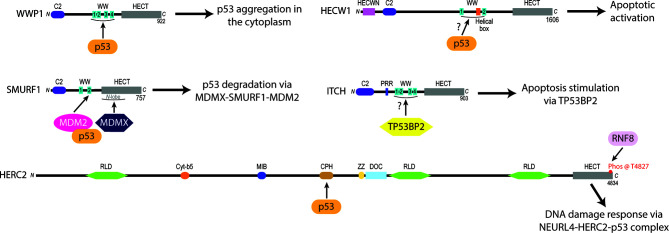
Experimentally identified p53 protein-protein interaction sites of certain NEDD4L and HERC subfamily HECT E3 ligases linked to oncogenesis. The WW domains of WWP1 are required to recruit p53 and induce its cytoplasmic aggregation (75). HECW1 uses an unknown domain to interact with p53 and upregulate apoptotic cellular activity (149). SMURF1 coordinates the heterodimerization of MDM2 and MDMX *via* its second WW domain and the *N*-lobe of its HECT domain to increase the MDM2-dependent K48 polyubiquitylation and subsequent degradation of p53 (78). ITCH is a NEDD4L subfamily E3 ligase that stimulates apoptotic pathways by the WW1-4 domain-dependent activation of Tumor protein p53-binding protein 2 (TP53BP2) (140, 141). HERC2 interacts with p53 through its CPH domain to monitor the p53-MDM2 feedback loop in NEURL4 DDR pathways (105).

WWP1 is a member of the NEDD4 subfamily that has been linked to colon, breast and oral cancers (31, 35, 39, 44, 152). WWP1 contains two WW domains that have been shown to recruit and modulate the activity of cancer related proteins like Runt-related transcription factor 2 (RUNX2) (153, 154), RING finger protein 11 (RNF11) (155, 156) and large tumor suppressor kinase 1 (LATS1) (157) *via* their proline-rich (PY) motifs (**Figure 1**). In addition to the interplay that occurs between WWP1 and these cancer-associated proteins, the enzyme can also interact with and regulate the activity of p53. Although p53 does contain a PY motif in its sequence (aa 68-91), WWP1 was observed to bind to p53’s DNA binding domain and not with its WW domains (**Figure 2**). Intriguingly, this association was abolished when the PY motif of p53 was deleted, suggesting that the conformation p53 adopts in the presence of its PY motif is required for proper WWP1-p53 complex formation (75). This unusual interaction was also found to increase the stability of p53, in contrast to the destructive effects mediated by binding of other ubiquitin ligases such as the RING E3 ubiquitin ligase MDM2 (158). The ubiquitylation activity of WWP1 was required for p53 stabilization, as an inactive version of WWP1 with its catalytic cysteine substituted with an alanine reduced p53 stability in a dominant negative fashion (75). Surprisingly, the WWP1-dependent stability of p53 was inversely proportional to the transcriptional activities of p53, which can attributed to the WWP1-p53 complex translocation from the nucleus to the cytoplasm and its subsequent aggregation (75).

The observation that WWP1 interacts with p53 suggests that WWP1 might be involved in tumor suppressor networks. For example, WWP1 silencing in two osteosarcoma cell lines promoted apoptosis and reduced cell invasion (159). This suppression also resulted in decreased expression of B-cell lymphoma 2 (Bcl2), matrix metallopeptidase 2 (MMP2), matrix metallopeptidase 9 (MMP9) and β-catenin, while pro-apoptotic proteins Bcl-2 associated X protein (Bax) and E-cadherin expression levels increased indicating that WWP1 may play a role in pro-apoptotic pathways (159). Studies have also demonstrated that WWP1 contributes to extrinsic apoptosis. For instance, the inhibition of WWP1 correlated with elevated levels of apoptosis initiator caspases 8 and 9, mitogen activated protein kinase 8 (MAPK8), as well as executioner caspase 7 *via* the TNR-related apoptosis-inducing ligand (TRAIL) death receptor (160). This change in phenotype was shown to be reversible with the overexpression of wild-type WWP1 but could not be rescued with an inactive version of the protein (160). Taken together, these results show that the ubiquitylation activity of WWP1 is required to inhibit apoptosis and promote the progression of particular colon and thyroidal cancers.

Studies have also demonstrated that WWP1 is involved in prostate cancer. For example, WWP1 overexpression caused by chromosomal duplication events was observed in prostate xenografts (31). Knockout studies also revealed that the loss of WWP1 resulted in increased transforming growth factor beta (TGF-β) receptor 1 (TβR-I) and mothers against decapentaplegic homolog 2 (Smad2) protein levels, which in turn enhanced the inhibitory effect of TGF-β (31). These results are consistent with previous studies highlighting the role of WWP1 as a negative regulator of TGF-β. In this regulatory pathway, WWP1 binds to Smad7 *via* a WW/PY interaction, independent of its ubiquitylation activity (161). This binding and regulation of Smad7 has also been observed with other members of the NEDD4 family (i.e. SMURF1 and SMURF2) (162–165). Co-immunoprecipitation experiments revealed that Smad7, WWP1 and TβR-I are in close proximity within the cell and may form a complex that allows for WWP1 to ubiquitylate TβR-1 and target it for proteasomal degradation (161).

These cumulative studies demonstrate that WWP1 is an important enzyme in the regulation p53 mediated gene transcription and apoptosis. With research that implicates WWP1 in prostate and osteosarcoma, it is critical that additional studies be conducted to investigate possibilities of modulating the activity of WWP1 to elicit specific anti-cancer responses in the cell. For example, by using structural techniques to determine how p53 is bound and stabilized by an active form of WWP1 in the cytoplasm, it will become possible to elucidate the biochemical and biophysical properties of the WWP1-p53 complex as well as the mechanism of ubiquitin transfer. This newfound knowledge will aid in the design of artificial molecular machinery that acts to repress WWP1 interactions with p53 and hence regain normal p53 anti-tumor activity in active cancer cells. Additionally, by expanding our knowledge of other important WWP1-substrate interactions, including the recognition and binding of WWP1 to TGF-β receptors, the successful elucidation and categorization of the WWP1 interactome can be achieved to provide a clearer map of the pathological role(s) of WWP1.

### HECW1 Positively Regulates p53 to Induce Apoptotic Pathway Activation

Recent biochemical studies have demonstrated that HECW1 possesses tumor suppressive activity by interacting with the *C*-terminus of p53 to upregulate the activation of p53-cisplatin dependent apoptotic cellular pathways (77, 149). This study also found that both the wild-type and isolated HECT constructs of HECW1 interact with p53, suggesting that the HECW1-dependent pro-apoptotic activation of p53 is independent of its ubiquitylation activity (77). Additionally, chromatin immunoprecipitation assays (ChIP) have demonstrated that HECW1 directs p53 to the p21wafi promoter region to induce the transcriptional activation of p53-related genes in response to carcinogenic cellular signals (149). There are currently no structural or mechanistic studies to explain how HECW1 forms a complex with p53 to regulate apoptotic anticancer activities within the cell. Taken together, these findings demonstrate the need for new structural and interactor-based studies on HECW1 to begin elucidating the exact mechanisms used by the enzyme to catalyze the activation of p53-induced apoptosis in cancerous cell lines. It will be important to determine the specific domains that HECW1 uses to recognize p53, define the conformational changes that HECW1 and p53 undergo to modulate p53 cellular activity, and decipher how the HECW1-p53 complex signals for the upregulation of p53 apoptotic signaling. Studies are also needed to examine how the HECW1-p53 interaction directs the migration of HECW1 to the nucleus where it promotes p53 activation of apoptotic related genes. By addressing these unknowns about the interplay between HECW1 and p53 cellular interplay, we may be able to fine tune the design of small molecule drugs that stimulate, activate, and enhance the HECW1-dependent activation of p53-induced apoptotic pathways in malignant cells.

### SMURF1-Dependent MDM2/MDMX Heterodimerization Negatively Regulates p53 Activity

SMURF1 is another member of the NEDD4 subfamily that acts to negatively regulate p53 activity during breast (40), ovarian (46), gastric (42), and glioblastoma (166) tumorigenesis by augmenting the ubiquitylation activity of MDM2 – a RING E3 ligase that specifically targets p53 for proteasomal degradation (76, 158, 167, 168). Previous findings have demonstrated that SMURF1 uses its WW domains to recognize and bind target substrates before carrying out their HECT-dependent ubiquitylation (169–171). Contrary to this traditional HECT E3 function, recent studies have discovered a unique function of SMURF1 whereby it promotes the cellular stability of MDM2 substrates by facilitating the heterodimerization of MDM2 and its homolog mouse double minute 4 protein (MDMX, aka MDM4) (78). The ability of SMURF1 to mediate MDM2-MDMX heterodimerization is thought to rely on the coordination of MDM2 with its second WW domain while MDMX interacts with the HECT *N*-lobe of SMURF1 (78) (**Figure 2**). A consequence of these multiprotein interactions is the structural inhibition of the MDM2 auto degradation pathway by MDMX. Not surprisingly, this interaction has been shown to result in the prolonged stability of MDM2 *in vivo* with an increase in the K48-polyubiquitylation activity of MDM2 on p53 (78).

To date, MDM2 and MDMX are the only substrates known to interact with the second WW domain and HECT domain of SMURF1 and not be targeted for ubiquitylation. It remains unresolved how the unusual stabilization effects that SMURF1 provides for MDM2 and MDMX occur on the mechanistic and molecular level. Of interesting note is that MDM2 and MDMX are bound by SMURF1 at its second WW domain and HECT domain, the same enzymatic regions used by the SMURF1 to carry out the ubiquitylation of its target substrates (76, 78, 158, 167, 168). These findings make it conceivable that SMURF1 might bind MDMX in an analogous fashion to an E2 cognate enzyme at the *N*-lobe of its HECT domain. Likewise, with MDM2 bound by SMURF1 by its second WW domain, it is possible that the coordination of MDM2 to MDMX is facilitated by conformational shifts in the SMURF1 domain architecture that are similar to the mechanisms used by the SMURF1 to carry out the ubiquitylation of its target substrates.

Recent studies have determined that MDM2 and MDMX form a ternary complex with SMURF1 to promote MDM2/MDMX heterodimerization, which subsequently can recruit p53 to the complex with MDM2 serving as the bridging molecule within the MDMX-SMURF1-MDM2-p53 multiprotein complex (76, 78). While the exact function of this ternary intermediate remains unclear in the context of p53 signaling, biochemical pull-down assays have shown that p53 does not associate with the MDMX-SMURF1-MDM2 heterotrimer if the *N*-terminus of MDM2 is truncated (a.a. 1-76) (78), and that SMURF1 does not interact with p53 in the absence from MDM2 (78). These findings suggest that the *N*-terminus of MDM2 functions to recognize and bind p53 in addition to being the point of interaction for the second WW domain of SMURF1. It is critical that follow up structural studies be performed to examine if the *N*-terminal domain of MDM2 causes any conformational changes within the MDMX-SMURF1-MDM2 ternary complex and how these conformational changes may play a role in MDM2-MDMX heterodimerization and p53 regulation. Likewise, it will be important to examine how the unique structural interactions that occur between SMURF1, MDM2, and MDMX impact the ability of SMURF1 to stabilize MDM2, and how the subsequent regulation of MDM2 activity by SMURF1 plays a role in p53-dependent cancer development.

SMURF1’s tight regulation of MDM2-dependent p53 ubiquitylation makes it a promising candidate for oncological drug development. An improved understanding of the mechanisms used by SMURF1 to promote MDM2-MDMX heterodimerization at the molecular level can be applied pharmacologically to regulate MDM2 p53 ubiquitylation activity in cancer cells, and therefore serve as a powerful tool to activate pro-apoptotic pathways and interrupt cell division.

## HERC2—A Novel HECT E3 Ubiquitin Ligase Linked to Cancer Development

Recent biochemical studies have identified HECT E3 ligases outside of the NEDD4 subfamily that regulate p53 activity. Here we describe how new research is beginning to reveal that HERC2 is a major player in mediating DNA repair by regulating p53 activity.

### HERC2 Mediates p53 Activity Through the Formation of a Multiprotein Ternary Complex in Response to DNA Damage

**Figure 3:**
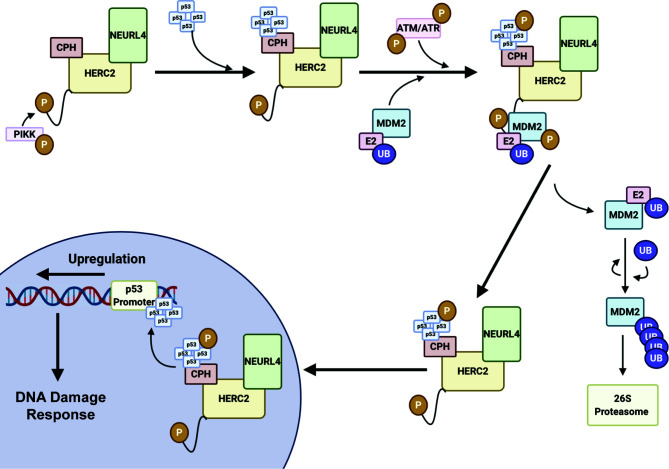
HERC2 serves as a master regulator of p53 gene transcription in response to DNA damage. HERC2 recruits oligomerized p53 with its CPH domain to form a NEURL4-HERC2-p53 ternary complex and is phosphorylated at T4827 on its C-terminal tail by phosphatidylinositol 3-kinase-like protein kinase (PIKK). The HERC2-NEURL4-p53 ternary complex coordinates with the RING E3 ubiquitin ligase MDM2. The kinases Ataxia telangiectasia mutated (ATM) and Ataxia telangiectasia and Rad3 related (ATR) are also recruited to the multiprotein structure. ATR and ATM carry out the phosphorylation of MDM2 and oligomerized p53. Phosphorylated MDM2 becomes unstable and dissociates from the HERC2 scaffolding to allow for its K48 auto-polyubiquitylation and the cytoplasmic stability of the HERC2-NEURL4-p53 ternary complex. The HERC2-NEURL4-p53 ternary complex migrates to the nucleus where it releases oligomerized p53. p53 binds to the p53 promoter regions where it initiates the upregulation of genes to aid in cellular DNA repair and the DNA damage response. This figure was created with BioRender.com.

HERC2 is a large 500 kDa multidomain E3 ubiquitin ligase that interacts with neuralized E3 ubiquitin protein ligase 4 (NEURL4) and MDM2 to modulate p53-dependent gene expression during the ATM and ATR induced DNA double strand break (DSB) repair response (105, 150). The HERC2-dependent activation of p53 is initiated by HERC2-NEURL4 complex formation that induces an allosteric conformational shift in the unique CPH domain of HERC2 (105, 150) (**Figure 3**). This change in conformation allows HERC2 to recruit oligomerized p53 with its CPH domain to form a NEURL4-HERC2-p53 ternary complex (105, 150). The NEURL4-HERC2-p53 complex then coordinates with the RING E3 ubiquitin ligase MDM2 that is usually responsible for targeting p53 for cytosolic trafficking *via* monoubiquitylation (172) and/or proteasomal degradation *via* K48-polyubiquitylation (57, 63, 64) under normal cellular conditions. However, during the ATM and ATR-activated DSB repair process, HERC2 is phosphorylated at T4827 on its *C*-terminal tail by phosphatidylinositol 3-kinase-like protein kinase (PIKK) to recruit the kinases ataxia telangiectasia mutated (ATM) and ataxia telangiectasia and Rad3 related (ATR) to the higher order NEURL4-HERC2-p53 complex where they catalyze the phosphorylation of oligomerized p53 and MDM2 (105, 150, 173). Following phosphorylation, MDM2 releases itself from the complex and carries out auto-polyubiquitylation to signal for its proteasomal degradation. Simultaneously, p53 is further stabilized by the CPH domain of HERC2 following its ATM and ATR-mediated phosphorylation and is no longer a target of MDM2 for polyubiquitylation (105, 150). The activated p53 oligomers are then transported by the HERC2-NEURL4 complex to the nucleus and bind to p53 promoter regions where p53-regulated genes including p53, p21 and MDM2 become upregulated to aid in cellular DNA repair (105, 150). Once the cell’s DNA damage response (DDR) is complete, ATM and ATR become targets of E3 ligases for proteasomal degradation and the HERC2-NEURL4 complex coordinates the MDM2-dependent degradation of p53 (167). Taken together, HERC2 acts as a master regulator of p53 transcriptional activation by selectively recruiting ATM and ATR kinases to modulate MDM2 and p53 stability throughout the DDR cycle. It remains unclear what specific structural conformations and mechanisms are used by HERC2 to control p53 stabilization by ATM and ATR dependent phosphorylation, or how p53 is targeted for degradation by MDM2-dependent ubiquitylation following its recruitment by the CPH domain of HERC2. Future studies are needed to clarify how the unique domains of HERC2 direct the ATM and ATR-dependent phosphorylation of p53 and p53-MDM2 regulation. An improved understanding of these mechanisms can potentially be exploited in novel oncological therapies that specifically target the p53-MDM2 feedback loop as a regulator of DNA replication and repair.

### HERC2 Is a Novel Oncogenic Suppresser That Regulates the DNA Damage Responses and Screens the Genome Prior to Replication

**Figure 4:**
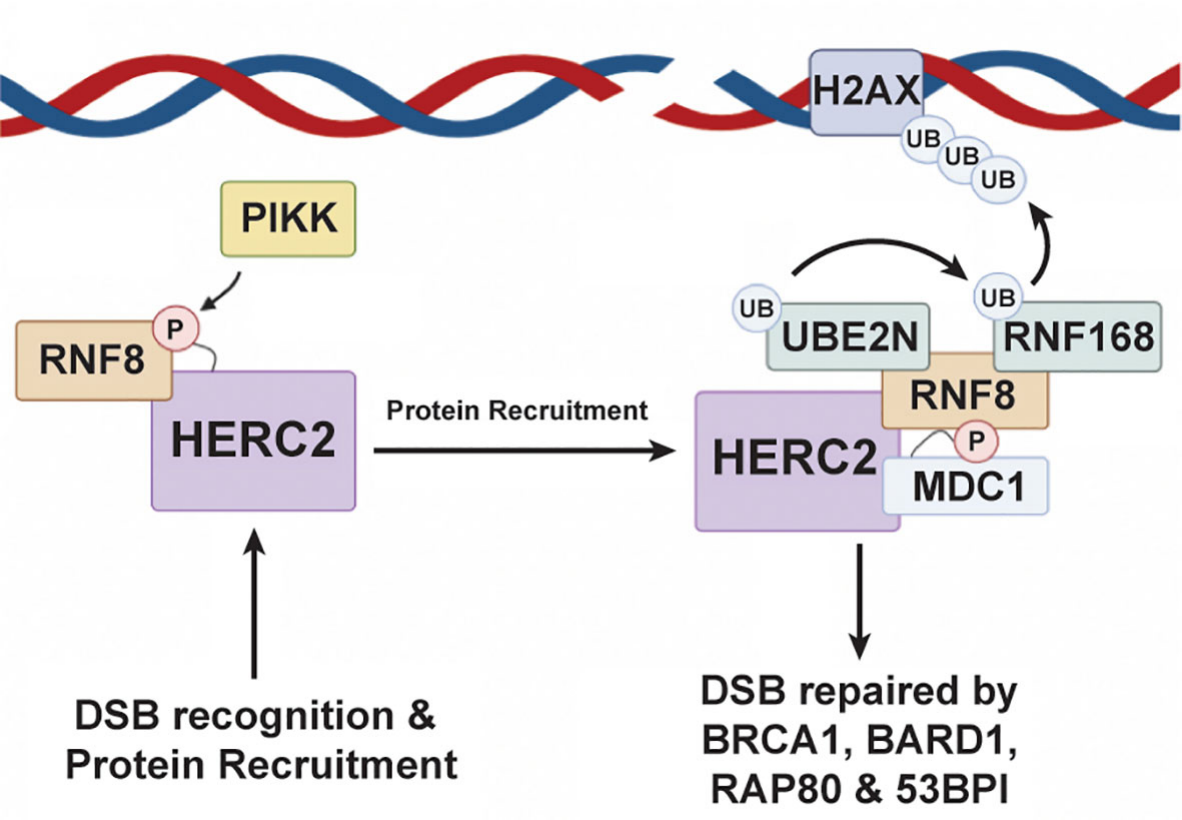
HERC2 serves as a scaffold to facilitate H2A ubiquitylation in response to DNA double strand breaks. HERC2 initiates the repair response for DSBs by using its catalytic HECT domain as a binding scaffold for RING finger protein 8 (RNF8), a RING E3 ubiquitin ligase, to bring RNF8 into close proximity to a site of DNA damage. After binding RNF8 to its HECT domain, HERC2 coordinates the formation of a complex between UBE2N (aka Ubc13), an E2 ubiquitin conjugating enzyme, and RNF168 to catalyze the attachment of K63-polyubiquitin chains onto histones at the site of damaged DNA. This HERC2-mediated K63-polyubiquitylation activity then signals for the recruitment of healing factors like breast cancer gene 1 (BRCA1), receptor associate protein 80 (RAP80), and 53BP1 to elicit an effective DDR response. This figure was created with BioRender.com.

In addition to regulating the MDM2-p53 transcriptional feedback loop, HERC2 can also prevent potentially oncogenic mutations from being passed into daughter cells by coordinating DNA double strand break (DSB) repair responses during the S and G2-M phases of mitosis (174–179). The HERC2-DSB repair pathway is initiated when a double strand break is sensed by the MRN complex – meiotic recombination 11 (MRE11), Nijmegen breakage syndrome 1 (NBS1), and radiation sensitive protein 50 (RAD50) (151). After recognizing the DSB, NBS1 recruits ATM kinases to the damage site where they phosphorylate Histone 2A Family Member X (H2AX) and Mediator of DNA Damage Checkpoint 1 (MDC1) (151). These phosphorylation events promote H2AX and MDC1 complexation and signal for the recruitment of HERC2 and RNF8, a RING E3 ubiquitin ligase, to the DSB (176, 180). Once arriving to the damage site, HERC2’s *C*-terminal tail is phosphorylated by PIKK at T4827 to promote its complexation with RING finger protein 8 (RNF8), an E3 ubiquitin ligase (**Figure 4**). Following RNF8 recruitment, HERC2 uses its *C*-terminal tail to stimulate the oligomerization of RNF8 and facilitates the formation of the HERC2-MDC1-RNF8 ternary complex. Phosphorylation signal cascades are used by these ternary complexes to carry out RNF8 mediated recruitment of RNF168, another RING E3 ubiquitin ligase, and its cognate E2 cognate enzyme UBE2N (aka Ubc13), to the DNA damage site (181). This multiprotein complex then works cooperatively to attach K63-polyubiquitin chains on to chromosomal histone proteins H2A and H2Ax. The K63 polyubiquitin linkages made on these histone sites are in close proximity to the DSB and serve as biochemical markers that recruit homologous DNA repair factors. These include breast cancer gene 1 (BRCA1), BRCA1 associated RING domain protein 1 (BARD1), receptor associated protein 80 (RAP80), and the non-homologous end joining repair factor 53BP1, all of which are required to carry out the full DDR response (176, 182, 183).

Recent studies indicate that HERC2 continues to play a role in the DSB repair pathway after the recruitment of these DNA repair factors. For example, it is suggested that HERC2 uses its phosphorylated *C*-terminal tail to help stabilize BRCA1, BARD1, and RAP80 by binding onto their degrons sites during the G2-M phase transition of cell replication thereby protecting these DDR proteins from proteasomal degradation while they carry out their DNA repair activities (176). This HERC2-dependent activity is critical to the prevention of cellular oncogenesis by acting to screen and repair the cell’s genetic material at potentially carcinogenic mutation sites that were overlooked during S-phase DNA replication.

Collectively, studies on the onco-suppressive activities of HERC2 suggest it as a promising drug target for future immunotherapeutic treatments, particularly in cases of breast cancer development and pathogenesis. While HERC2 has been extensively characterized in a biochemical context, the mechanistic and structural basis for the involvement of HERC2 in the NEURL4-p53-MDM2 mediated DSB and DDR pathways remain largely unexplored. Intriguingly, in both pathways HERC2 serves as a scaffold that recruits and orchestrates the timely activities of regulatory proteins that are key to regulating p53-MDM2 intracellular concentration and/or DNA integrity. It is conceivable that HERC2 targeted drug development needs to be focused on enhancing the onco-suppressive activities of HERC2 to control the progression of cancer. As a prerequisite to developing these treatments, it will be paramount that structural studies be conducted to learn how HERC2 uses its different protein-protein interaction domains, including its catalytic HECT domain, to carry out specific molecular mechanisms that regulate p53 activity and the DDR response. For example, there are many unanswered questions on how the *N*-terminal variable domains of HERC2 contribute to substrate recognition and HERC2-dependent ubiquitylation. The mechanisms used by HERC2 in DNA maintenance/repair and p53-MDM2 modulation in the cell are also unknown. Additionally, studies on the role of conformationally flexible in the acidic *C*-terminal tail of HERC2 for building polyubiquitin chains during DDR and p53 oligomerization and clarifying how HERC2 recognizes and targets proteins to the p53 promoter region to regulate p53-related gene expression and/or a damaged DNA site to facilitate DNA repair are needed. Expanded studies on HERC2 could prove to be pivotal in the development of new immunotherapeutic treatments that target specific HECT E3 protein-protein interactions in the cell to elicit a specific intracellular immunological response against cancers.

## HECT E3 Ubiquitin Ligases as Cancer Drug Targets—Moving Forward

Recent advances in the biochemical and structural characterization of HECT E3 ubiquitin ligases have revealed these enzymes are critical regulators of the p53-MDM2 and DDR pathways. To date, only one HECT E3 ubiquitin ligase specific cancer drug, Bortezomib, has been reported to effectively modulate the activity of the NEDD4L and HERC subfamily ligases discussed in this review (184). Intriguingly, a recent 2019 study used phage library analysis to identify a class of bicyclic peptides that demonstrate a general inhibitory effect on the HECT E3 ligases ITCH, WWP1, SMURF1 and HECW1 by competitively binding to the E2 interaction site on the *N*-lobe of the HECT domain (185). However, the activity of these small molecule competitive inhibitors provided no specific anticancer effects when tested in tumorigenic cell lines (185). Perhaps one of the largest obstacles for developing HECT specific anticancer therapeutics is the diversity of mechanisms and structures associated within each HECT subfamily and the reality that many of these mechanisms, structures, and their functional roles in cancer pathogenesis remain largely unknown. Concurrently, the amount of knowledge that remains to be uncovered on the NEDD4L subgroup, as well as other members of the broader HECT family, provide many opportunities for the generation of novel therapeutics to treat a broad range of cancers. As new discoveries continue to be made on this fascinating group of proteins, our knowledge into the scope of molecular mechanisms, protein-protein interactions, and identified substrates engaged by HECT E3 ubiquitin ligases in oncogenesis will continue to expand.

It will become increasingly important that new structural and biophysical studies be conducted on members of the HECT E3 ubiquitin ligases to clarify how the HECT domain architecture contributes to HECT catalytic dysfunction in p53 and related cellular pathways. For example, further examination of the mechanisms used by SMURF1 to catalyze MDM2/MDMX heterodimerization and increase the MDM2-dependent ubiquitylation of p53 could allow for the synthesis of small molecule inhibitors drugs that block MDMX-SMURF1 complex formation. Likewise, the development of therapies that promote the upregulation of p53 pro-apoptotic cellular signaling in SMURF1 overexpressed cells to disrupt tumor growth is another avenue that needs to be studied. An improved molecular understanding of how HERC2 uses its multidomain structure to direct the ATM and ATR-dependent phosphorylation of p53 and MDM2 will be pivotal to uncovering the HERC2-dependent mechanisms involved with p53-MDM2 regulation. Expanded studies on HECW1 and WWP1 will also be critical in the development of small molecule therapeutics to target these enzymes and their roles in p53 regulation. With so little known about the diverse structural, molecular, and mechanistic bases used by HECT E3 ligases to regulate the p53-MDM2, DDR, and other pathways implicated in oncogenesis, now is an exciting time to be researching this class of enzymes that are at the nexus for the development of new onco-therapeutic treatments.

## Author Contributions

Conceptualization, NM, RL, and DS. Writing—original draft preparation, review and editing, and revisions, NM, RL, and DS. Figures, NM, RL, and DS. Supervision, DS. All authors contributed to the article and approved the submitted version.

## Funding

This research was supported by the National Institutes of Health, R15GM126432 (DS). NM was the recipient of an Arthur E. Martell and Thomas T. Sugihara Summer Undergraduate Scholarship from Clark University.

## Conflict of Interest

The authors declare that the research was conducted in the absence of any commercial or financial relationships that could be construed as a potential conflict of interest.

## Abbreviations

APC, Anaphase promoting complex; ATM, Ataxia telangiectasia mutated kinase; ATR, Ataxia telangiectasia and Rad3 related kinase; BARD1, BRCA1 associated RING domain protein 1; Bax, Bcl-2 associated X protein; Bcl2 - B-cell lymphoma 2; BRCA1, Breast cancer gene 1; ChIP, Chromatin immunoprecipitation assays; CPH, Cullin-7-PARC-HERC2 domain; Cyt-b5, Cytochrome-b5 like domain; DDR, DNA damage response; DOC, Downregulated in ovarian cancer domain; DSB, Double strand break; E1, Ubiquitin activating enzyme; E2, Ubiquitin conjugating enzyme; E3, Ubiquitin ligase; H2AX, Histone 2A family member X; HECT, Homologous to E6AP C-terminus; HECW1, HECT, C2, and WW-domain containing protein 1; HECW2, HECT, C2, and WW-domain containing protein 2; HERC, HECT and RLD containing; HERC2 - HECT and RLD containing protein 2; LATS1, Large tumor suppressor kinase 1; MAPK8, Mitogen activated protein kinase 8; MDC1, Mediator of DNA damage checkpoint 1; MDM2, Mouse double minute 2; MDMX, Mouse double minute 4 (aka MDM4); MIB, Mind bomb domain; MMP2, Matrix metallopeptidase 2; MMP9, Matrix metallopeptidase 9; MRE11, Meiotic recombination 11; NBS1, Nijmegen breakage syndrome 1; NEDD4, Neuronal precursor cell-expressed developmentally downregulated 4; NEURL4, Neuralized E3 ubiquitin protein ligase 4; p53, Tumor suppressor protein p53; PIKK, Phosphatidylinositol 3-kinase-like protein kinase; PPxY or PY, proline-rich motif; PTM, Post translational modification; RAD50, Radiation sensitive protein 50; RAP80, Receptor associated protein 80; RBR, RING-between-RING, RING-BRcat-Rcat; RCD, Regulatory cell death; RING, Really interesting new gene; RLD, Regulator of chromosome condensation 1-like domain; RNF11, RING finger protein 11; RNF8, RING finger protein 8; RUNX2, Runt-related transcription factor 2; Smad2, Mothers against decapentplegic homolog 2; Smad7, Mothers against decapentplegic homolog 7; SMURF1, SMAD ubiquitylation regulatory factor 1; SMURF2, SMAD ubiquitylation regulatory factor 2; TGF-β, Transforming growth factor beta; TRAIL, TNR-related apoptosis-inducing ligand; TβR-I, TGF-β receptor I; UBE2N, Ubiquitin conjugating E2 enzyme UBE2N (aka Ubc13); WW, Tryptophan-tryptophan domain; WWP1, WW-domain containing protein 1; WWP2, WW-domain containing protein 2; ZF, Zinc finger.
